# Analgesic Mechanisms of Antidepressants for Neuropathic Pain

**DOI:** 10.3390/ijms18112483

**Published:** 2017-11-21

**Authors:** Hideaki Obata

**Affiliations:** Center for Pain Management and Department of Anesthesiology, Fukushima Medical University, 1 Hikarigaoka, Fukushima-City, Fukushima 960-1295, Japan; obata@fmu.ac.jp; Tel.: +81-24-547-1533

**Keywords:** noradrenaline, 5-HT, dopamine, spinal cord, α_2_-adrenergic receptors, locus coeruleus, spinal nerve ligation, hyperalgesia, allodynia, rats

## Abstract

Tricyclic antidepressants and serotonin noradrenaline reuptake inhibitors are used to treat chronic pain, such as neuropathic pain. Why antidepressants are effective for treatment of neuropathic pain and the precise mechanisms underlying their effects, however, remain unclear. The inhibitory effects of these antidepressants for neuropathic pain manifest more quickly than their antidepressive effects, suggesting different modes of action. Recent studies of animal models of neuropathic pain revealed that noradrenaline is extremely important for the inhibition of neuropathic pain. First, increasing noradrenaline in the spinal cord by reuptake inhibition directly inhibits neuropathic pain through α_2_-adrenergic receptors. Second, increasing noradrenaline acts on the locus coeruleus and improves the function of an impaired descending noradrenergic inhibitory system. Serotonin and dopamine may reinforce the noradrenergic effects to inhibit neuropathic pain. The mechanisms of neuropathic pain inhibition by antidepressants based mainly on experimental findings from animal models of neuropathic pain are discussed in this review.

## 1. Introduction

Although antidepressants were not originally designed to act as analgesics, they are reported to have analgesic effects for chronic pain. Antidepressants have virtually no antinociceptive effects, but are considered first-line drugs of choice for neuropathic pain [1,2,3,4] and treatment of fibromyalgia [5]. Specific antidepressants with analgesic effects include tricyclic antidepressants (TCA), which have long been used, and serotonin noradrenaline reuptake inhibitors (SNRI), which are comparatively new antidepressants. Selective serotonin reuptake inhibitors (SSRI), which are frequently used to treat depression, are not effective against chronic pain [1,2,3].

## 2. Effects of Antidepressants on Neuropathic Pain Differ from Their Effects on Depression

Antidepressants, along with pregabalin/gabapentin (voltage-dependent calcium channels α2δ subunit ligands) are used as first-line drugs for treating neuropathic pain [1,2,3,4]. Psychologic problems play an important role in chronic pain. Protracted pain causes anxiety accompanied by a progressive depressive state and enhanced pain sensations. Therefore, antidepressant medications may be effective against chronic pain by their effects to improve the depressive state. Antidepressants inhibit neuropathic pain, however, even when the patient is not in a depressive state [6]. In addition, the effects of antidepressants on depression characteristically take approximately two to four weeks to be observed from the time the drug is first taken, whereas the analgesic effects on chronic pain manifest in as little as few days to one week [7]. Therefore, the analgesic effects of antidepressants on chronic pain likely involve a mechanism different from that mediating their antidepressive effects.

## 3. Noradrenaline Is Extremely Important for Inhibiting Neuropathic Pain

The pharmacologic effects of antidepressants involve binding to noradrenaline and serotonin (5-HT) transporters. Reuptake of these neurotransmitters is i nhibited, leading to increased levels of noradrenaline and 5-HT in the synaptic cleft [8,9,10,11]. What type of antidepressants is most effective against neuropathic pain? The “number needed to treat” (NNT) is an index used to compare the efficacy of medications based on the results obtained in a variety of clinical studies (meta analysis) and is represented as the number of patients treated for whom pain was reduced by up to 50%, with a smaller numerical value indicating a stronger efficacy [12,13]. According to a report from Finnerup et al., the NNT of TCA to inhibit peripheral neuropathic pain is approximately 2–3. The NNT of dual-type TCAs (e.g., amitriptyline, imipramine, clomipramine), which inhibit reuptake of both noradrenaline and 5-HT, is 2.1. The NNT for noradrenaline reuptake inhibitors (nortriptyline, desipramine) is approximately 2.5 [14]. The NNT for SNRI is 5.0 and for SSRI is 6.8 in painful polyneuropathy [2]. Based on these results, an antidepressant that inhibits reuptake of both noradrenaline and 5-HT has stronger analgesic effects than a drug that selectively inhibits reuptake of only one of these neurotransmitters, and noradrenaline plays a greater role than 5-HT in the analgesic action.

## 4. Noradrenaline Inhibits Neuropathic Pain in the Spinal Cord

**Figure 1:**
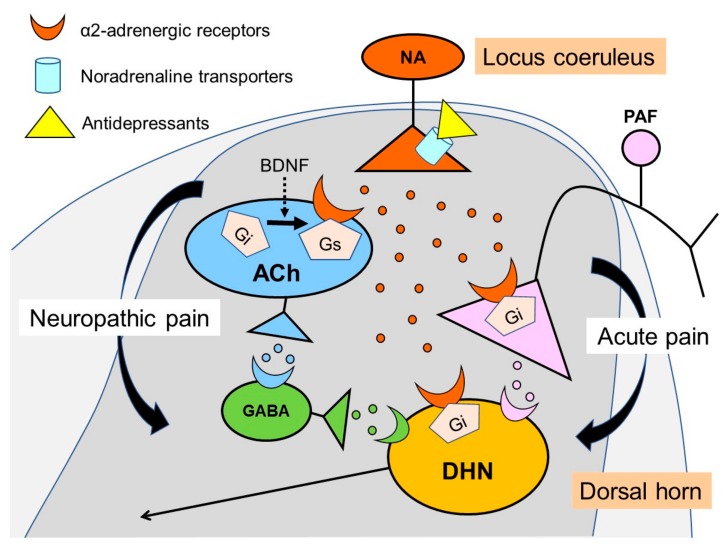
Schematic illustration of analgesic effects of antidepressants and noradrenaline in the dorsal horn of the spinal cord. Antidepressants increase noradrenaline via blocking of noradrenaline transporters at the terminal of the descending noradrenergic fiber from the locus coeruleus. Noradrenaline inhibits acute pain through α_2_-adrenergic receptors by pre-synaptic (inhibit neurotransmitters release) and post-synaptic (hyperpolarize cell membranes) mechanisms. In neuropathic pain states, however, α_2_-adrenergic receptors in the cholinergic interneurons change from inhibitory to excitatory through G-protein switch (from Gi to Gs) by the effect of brain-derived neurotrophic factor (BDNF) increasing after nerve injury. Released acetylcholine bind to muscarinic receptors, by which produce analgesia thorough GABA release. PAF; primary afferent fibers, NA; noradrenaline, DHN; dorsal horn neurons, ACh; acetylcholine, Red circle; noradrenaline, Blue circle; acetylcholine, Green circle; GABA, Pink circle; excitatory neurotransmitters.

Noradrenaline reuptake inhibition enhances analgesic effects, mainly through α_2_-adrenergic receptors in the dorsal horn of the spinal cord. The α_2_-adrenergic receptors are coupled to the inhibitory G protein (Gi/o), which inhibits the presynaptic voltage-gated Ca^2+^ channels in the dorsal horn of the spinal cord that inhibits the release of excitatory neurotransmitters from primary afferent fibers. At the same time, G protein-coupled inwardly rectifying K^+^ channels are opened on the post-synaptic spinal cord dorsal horn cells, the cell membranes are hyperpolarized, and excitability is decreased [15]. While activation of the α_2_-adrenergic receptors of the spinal cord dorsal horn has weak antinociceptive effects against noxious stimuli, extensive research indicates that it is extremely effective against allodynia and hyperalgesia associated with neuropathic pain [16,17]. The reason for the increasing efficacy for hypersensitivity of spinal α_2_-adrenergic receptors stimulation is that nerve injury changes the function of α_2_-adrenergic receptors in the dorsal horn of the spinal cord [18,19], while at the same time the interaction with the cholinergic interneurons strengthens [20,21,22,23]. Our findings support the importance of α_2_-adrenergic receptors in the spinal cord dorsal horn for the inhibition of neuropathic pain. We used a rat model of neuropathic pain known as spinal nerve ligation (SNL). In this procedure, the L5 spinal nerve is ligated on one side [24]. When mechanical pressure is applied to the ipsilateral hind paw on the ligated side using a paw-pressure test, the withdrawal threshold decreases and mechanical hyperalgesia develops. Intrathecal administration of an α_2_-adrenergic receptor agonist, dexmedetomidine, leads to the release of acetylcholine to the spinal cord and mechanical hyperalgesia is inhibited through the muscarinic receptors [16]. In animals with nerve injury, the α_2_-adrenergic receptors expressed in the cholinergic interneurons of the spinal cord dorsal horn are coupled with excitatory G protein (G_S_) by the action of brain-derived neurotrophic factor (BDNF) through TrkB receptor and acetylcholine is released by stimulation of the α_2_ adrenergic receptors [23]. As a result, muscarinic receptors, which induce gamma-aminobutyric acid (GABA) release [25,26], contribute to the inhibitory effects of α_2_-adrenergic receptor activation for neuropathic pain (Figure 1). Thus, the pain relief mediated by noradrenaline in the dorsal horn of the spinal cord is more effective for neuropathic pain than for nociceptive pain due to plastic changes of the α_2_-adrenergic receptors.

**Figure 2:**
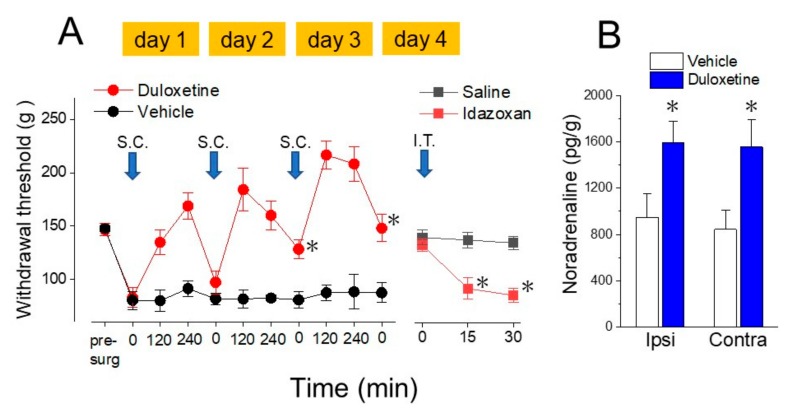
Antihyperalgesic effects of duloxetine in rats after nerve injury by noradrenaline increase in the spinal cord. (**A**) Effects of three daily injections of duloxetine on hind paw mechanical hyperalgesia after spinal nerve ligation (SNL) in rats. Rats were administered duloxetine (10 mg/kg/day, subcutaneous injection) or vehicle for three consecutive days. Each day, withdrawal thresholds in ipsilateral hind paw were measured at time 0 (baseline: before each duloxetine injection), and 120 and 240 min after the injection. Three daily treatment increased baseline withdrawal thresholds (time 0) of on days 3 and 4 than on day 1 (* *p* <0.05 compared with the duloxetine group at time 0 on day 1). Acute antihyperalgesic effects until 240 min after injection were also observed. Then, rats were injected intrathecally (i.t.) with idazoxan, an α_2_-adrenoceptor antagonist (30 μg/20 μL) or vehicle. The injection of idazoxan reversed the antihyperalgesic effect of duloxetine (* *p* <0.05 compared with the vehicle group at each time-point). (**B**) Spinal cord tissue from SNL rats injected with duloxetine (10 mg/kg/day) or vehicle for three consecutive days was homogenized, and the noradrenaline content was measured. Noradrenaline was increased with duloxetine treatment at both ipsilateral and contralateral to the SNL compared to the vehicle group (* *p* <0.05). Data in this figure published from Ref. [27].

Intraperitoneal administration of duloxetine, an SNRI, to SNL rats increases the withdrawal threshold for at least 4 h, but the effect disappears after 24 h. When duloxetine is administered for three consecutive days, the withdrawal threshold gradually increases and returns to pre-SNL levels. This increase in the withdrawal threshold is reversed by intrathecal injection of idazoxan, an α_2_ adrenergic receptor antagonist, and the amount of noradrenaline in the dorsal horn of the spinal cord increases after three daily injections of duloxetine [27] (Figure 2). In addition, in animals pretreated with a noradrenergic neurotoxin (DSP-4), the antihyperalgesic effect of duloxetine is weakened [28]. Intraperitoneal administration of amitriptyline, a TCA, over consecutive days gradually increases the withdrawal threshold, but this antihyperalgesic effect is reversed by intrathecal injection of an α_2_-adrenergic antagonist [28].

**Figure 3:**
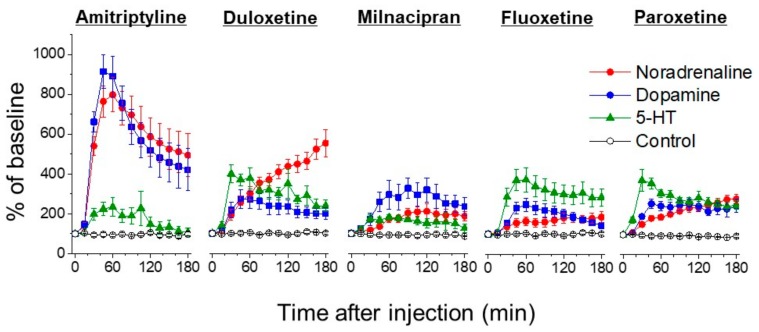
Percentage changes of noradrenaline, dopamine, and 5-HT levels in the dorsal horn of the lumbar spinal cord in rats after intraperitoneal injection of 10 mg/kg of amitriptyline (TCA), duloxetine (SNRI), milnacipran (SNRI), fluoxetine (SSRI) and Paroxetine (SSRI). Under isoflurane anesthesia, microdialysis studies ware performed after thoracolumbar laminectomy, and monoamines were measured by using high-performance liquid chromatography with electrochemical detection. All monoamines were increased after injection of all antidepressants (all *p* <0.05 compared to control; saline or vehicle, by two-way repeated-measures analysis of variance. Some data in this figure published from Ref. [32]).

Noradrenaline in the dorsal horn of the spinal cord is increased by a single intraperitoneal injection of antidepressants such as amitriptyline (TCA), duloxetine and milnacipran (SNRI), or fluoxetine and paroxetine (SSRI) (Figure 3). In addition, a single administration of paroxetine produces an anti-hyperalgesic effect, which is inhibited by intrathecal injection of an α_2_-adrenergic receptor antagonist [29] Fluoxetine and paroxetine have weak inhibitory effects on noradrenaline transporters [30,31], and thus their effects to increase noradrenaline are likely indirect. These findings suggest that the increase in noradrenaline in the spinal cord plays a crucial role in the inhibitory effects of antidepressants on neuropathic pain.

## 5. Actions of Antidepressants on the Locus Coeruleus

The locus coeruleus (LC) comprises a group of nerve cells containing noradrenaline located bilaterally in the brain. The LC has the greatest amount of noradrenaline in the central nervous system and is located on the right and left of the posterior brainstem facing the fourth ventricle [33,34]. Noradrenergic nerve fibers project virtually throughout the entire central nervous system and play a role in sleep, wakefulness, cognition, learning and stress in the brain [35,36,37]. In the spinal cord, noradrenergic nerve fibers regulate endogenous analgesia, posture and motion, the autonomous nervous system, and other vital functions [38,39,40]. The neuronal activity of the LC is characterized by a tonic (autonomous activity) mode and a phasic (activity that reacts to stimulus) mode. Phasic activity is an excitatory reaction within a short period of time in which stimuli induce the release of an excitatory amino acid (mainly glutamic acid) in the LC. Phasic activity during the tonic activity mode at a medium level from a lower level plays an important role in attention, movement and concentration on outside stimuli, such as cognitive functions, endogenous analgesia and a variety of other vital functions [35,41]. Descending noradrenergic neurons are an extremely important mechanism of endogenous analgesia. In rats, the bilateral LC is excited phasically by noxious stimulation, and releases noradrenaline through projections to the bilateral spinal cord dorsal horn [38,41,42].

How does the activity of the descending noradrenergic inhibitory system from the LC change in a neuropathic pain state? Noxious stimulation induced analgesia (NSIA) is an animal model in which the intensity of endogenous analgesia can be measured. The withdrawal threshold in response to the mechanical stimulus on the hind paw greatly increases after forepaw capsaicin injection by activation of the endogenous analgesia [43]. An increase in noradrenaline in the spinal cord affects the NSIA [41,43,44]. This means that the LC is activated phasically due to the pain induced by the capsaicin, and the noradrenaline that is released to the spinal cord mediates antinociceptive effects through α_2_-adrenergic receptors. When the same experiment was carried out using animal models of neuropathic pain (SNL animals), the NSIA was no longer recognized six weeks after nerve injury (an increase in the withdrawal threshold of the hind paw no longer occurred when capsaicin was administered to the forepaw), and the noradrenaline is not increased in the spinal cord [27,41,44]. At this time, the tonic nerve activity of the LC increased due to nerve injury, but the phasic reactivity to the noxious stimuli disappeared [41]. Based on these results, the phasic activity of the neuronal cells of the LC gradually declined in the neuropathic pain model after a long period of time had passed from the nerve injury, and the descending noradrenergic inhibitory system was impaired.

In animals with nerve injury and impaired NSIA, administering duloxetine and amitriptyline over several consecutive days recovers the NSIA [27,44]. Although the increase in noradrenaline in the spinal cord induced by these antidepressants plays a part in the NSIA recovery, it is possible that the effect of the drugs on the LC contributed to the effect. The LC receives inputs from a variety of sites of the central nervous system and its activity is controlled by both noradrenaline and 5-HT [45]. Antidepressants increase noradrenaline around the LC [46], and inhibit the activity of the LC through α_2_- adrenergic receptors [47,48]. In contrast, another report suggests that when duloxetine and desipramine are administered consecutively, the increased noradrenaline desensitizes the α_2_-adrenergic receptors in the LC [49]. In animal models of neuropathic pain, the reaction of the LC to noxious stimuli differs from that in normal animals due to sensitization via *N*-methyl-d-aspartic acid (NMDA) receptors, but this report indicates that the reaction is normalized by the consecutive administration of duloxetine and desipramine [49].

Furthermore, a previous study demonstrated that nerve injury increases the basal extracellular glutamate concentration in the LC [41], which may reduce noxious stimulation-evoked glutamate release, thereby diminishing α-amino-3-hydroxy-5-methyl-4-isoxazoleproprionic acid (AMPA) receptor-mediated LC activation, which is important for inducing NSIA. Another previous study demonstrated that antidepressants increased BDNF levels in astrocyte cultures [50]. BDNF triggers AMPA receptor GluA1 phosphorylation and regulates trafficking of the AMPA receptor to the cell membrane [51]. Therefore, antidepressants may reactivate impaired LC function after nerve injury by increasing BDNF levels.

## 6. The Role of 5-HT

Many antidepressants block 5-HT transporters, leading to increased 5-HT in the synaptic cleft. The role of 5-HT on the inhibitory effects of antidepressants against neuropathic pain, however, is unclear. Although SSRIs are popular drugs for the treatment of depression, they are not recommended for the treatment of neuropathic pain [1,2,3]. Despite some reports of their effectiveness in randomized controlled trials in patients with chronic pain, the NNT for SSRIs is higher than that for TCA and SNRI [2,14]. For this reason, 5-HT is thought to play a less important role than noradrenaline in the inhibition of neuropathic pain, but simultaneous administration of noradrenaline and 5-HT selective reuptake inhibitors in an animal experiment produce a synergistic analgesic effect [52], suggesting that 5-HT has auxiliary actions.

5-HT in the dorsal horn of the spinal cord greatly contributes to pain modulation. Inhibitory 5-HT_1A_ receptors, and excitatory 5-HT_2A/2C_, 5-HT_3_, and 5-HT_7_ receptors that strongly contribute to nociceptive transmission are expressed in the dorsal horn of the spinal cord [53,54,55,56]. These receptors are present in the pre-synaptic terminals of primary afferent nerve fibers, inhibitory interneurons, excitatory interneurons and projection neurons, and modify nociceptive transmission. When the pain sensation reaches the brain, the descending inhibitory system is mobilized from a variety of sites in the cerebral cortex and activates the periaqueductal gray (PAG) [56,57]. The PAG closely controls the rostroventromedial medulla (RVM) and modifies pain via projecting fibers from the RVM to the dorsal horn of the spinal cord [56,57,58]. The RVM includes the nucleus raphe magnus, which projects abundant serotonergic fibers to the spinal cord dorsal horn [58]. Descending serotonergic projections from the RVM to the spinal cord dorsal horn exert both inhibitory and facilitatory effects on pain processing depending on the pain state, acute or chronic [58,59,60]. In neuropathic pain models, many studies reported that ablation of descending 5-HT pathways inhibit pain hypersensitivity [61,62,63], and have also demonstrated that nerve injury induces descending facilitation by activating spinal 5-HT_3_ receptors [64,65]. Activation of descending serotonergic neurons form the RVM is required, however, for this descending facilitation to occur. In contrast, direct intrathecal injection of 5-HT or a 5-HT_3_ agonist inhibits allodynia in a rat neuropathic pain model [66,67]. Systemic administration of paroxetine, an SSRI, produces an anti-hyperalgesic effect in a rat model of neuropathic pain through spinal 5-HT_3_ receptors [29], because the drug directly increases 5-HT in the spinal cord by inhibiting 5-HT transporters.

Several lines of evidence suggest that 5-HT receptor function changes in neuropathic pain states. Although 5-HT_2A_ receptors in the spinal cord dorsal horn contribute to the suppression of neuropathic pain [68,69,70], the inhibitory effects of systemically administered SSRIs on hyperalgesia after nerve injury are stronger when spinal 5-HT_2A_ receptors are disrupted from their associated PDZ proteins by intrathecal injection of a peptide (TAT-2ASCV) [71]. Systemic administration of a 5-HT_1A_ receptor agonist (NLX-112) strongly inhibits inflammatory pain, but is less effective against neuropathic pain [72].

Thus, there are many reports suggesting a less important role for 5-HT in inhibition of neuropathic pain compared to acute pain. Increased 5-HT in the spinal cord by antidepressants may, however, play some inhibitory role for neuropathic pain.

## 7. The Role of Dopamine

Descending dopaminergic neurons, which project from the mesolimbic A11 dopamine cell group to the spinal cord dorsal horn, release dopamine in the dorsal horn of the spinal cord and inhibit nociceptive transmission by mediating dopamine D2-like receptors [73,74]. Buproprion, a dopamine noradrenaline reuptake inhibitor, increases noradrenaline and dopamine levels in the spinal cord and suppresses hyperalgesia in a rat neuropathic pain model through α_2_-adrenergic receptors and D2-like receptors [75]. No other antidepressants are reported to strongly inhibit dopamine transporters. Nevertheless, we demonstrated that intraperitoneal administration of amitriptyline (TCA), duloxetine and milnacipran (SNRI) and fluoxetine (SSRI) at a dose of 10 mg/kg, all increased dopamine in the spinal cord and inhibited hyperalgesia in a rat model of neuropathic pain through D2-like receptors [32]. There are few dopamine transporters in the frontal cortex and reuptake of dopamine is mediated by noradrenaline transporters [76]. It is unclear why antidepressants increase dopamine levels in the spinal cord.

Dopaminergic neurons in the ventral tegmental area (A10) release dopamine in the nucleus accumbens and enhance analgesic activity through D2-like receptors [77,78,79]. A previous study demonstrated that duloxetine (25 mg/kg orally) increases dopamine in the nucleus accumbens, but amitriptyline and maprotiline (both 50 mg/kg orally) do not have this effect [80]. Dopamine enhances the antinociceptive effects in the striatum [81,82]. Although it is unclear whether antidepressants increase dopamine in these brain areas, dopamine increases in the central nervous system are likely to play a role in the inhibitory effect of antidepressants on neuropathic pain.

## 8. Other Actions

Antidepressants have a number of other actions in addition to increasing monoamines that may contribute to the inhibition of neuropathic pain. First, they act as sodium channel blockers [83,84]. Sodium channel blockers inhibit ectopic discharges occurring when there is nerve damage, thereby inhibiting neuropathic pain [85,86]. Second, some antidepressants act as NMDA receptor antagonists [87,88]. NMDA receptors, which are expressed in the neurons of the dorsal horn of the spinal cord, induce wind-up and central sensitization result in contribute to the onset and maintenance of neuropathic pain [89,90].

TCAs also act as α_1_-adrenergic receptors [91], calcium channel blockers [92], potassium channel activators [93], modulate the adenosine system [94] and increase GABA-B receptor function [95]. They activate opioid receptors [96], inhibit the production of nitric oxide, prostaglandin E2 [97] and have a variety of other actions that may inhibit neuropathic pain in a complex manner.

## 9. Conclusions

The main mechanism of antidepressants that inhibit neuropathic pain is first, to increase noradrenaline in the spinal cord, and second, to act on the LC, thereby directly inhibiting pain and activating the impaired descending noradrenergic inhibitory system. Dopamine and 5-HT also increase in the central nervous system and may enhance the inhibitory effects of noradrenaline in an auxiliary manner.

## References

[1] Finnerup N.B., Attal N., Haroutounian S., McNicol E., Baron R., Dworkin R.H., Gilron I., Haanpää M., Hansson P., Jensen T.S. (2015). Pharmacotherapy for neuropathic pain in adults: A systematic review and meta-analysis. Lancet Neurol..

[2] Finnerup N.B., Sindrup S.H., Jensen T.S. (2010). The evidence for pharmacological treatment of neuropathic pain. Pain.

[3] Attal N., Cruccu G., Baron R., Haanpää M., Hansson P., Jensen T.S., Nurmikko T. (2010). EFNS guidelines on the pharmacological treatment of neuropathic pain: 2010 revision. Eur. J. Neurol..

[4] Dworkin R.H., O’Connor B., Backonja M., Farrar J.T., Finnerup N.B., Jensen T.S., Kalso E.A., Loeser J.D., Miaskowski C., Nurmikko T.J. (2007). Pharmacologic management of neuropathic pain: Evidence-based recommendations. Pain.

[5] Calandre E.P., Rico-Villademoros F., Slim M. (2015). An update on pharmacotherapy for the treatment of fibromyalgia. Expert Opin. Pharmacother..

[6] Fishbain D.A., Cutler R.B., Rosomoff H.L., Rosomoff R.S. (1998). Do antidepressants have an analgesic effect in psychogenic pain and somatoform pain disorder? A meta-analysis. Psychosom. Med..

[7] Onghena P., van houdenhove B. (1992). Antidepressant-induced analgesia in chronic non-malignant pain: A meta-analysis of 39 placebo-controlled studies. Pain.

[8] Micó J.A., Ardid D., Berrocoso E., Eschalier A. (2006). Antidepressants and pain. Trends Pharmacol. Sci..

[9] Dharmshaktu P., Tayal V., Kalra B.S. (2012). Efficacy of antidepressants as analgesics: A review. J. Clin. Pharmacol..

[10] Berton O., Nestler E.J. (2006). New approaches to antidepressant drug discovery: Beyond monoamines. Nat. Rev. Neurosci..

[11] Sindrup S.H., Otto M., Finnerup N.B., Jensen T.S. (2005). Antidepressants in the treatment of neuropathic pain. Basic Clin. Pharmacol. Toxicol..

[12] McQuay H.J., Tramèr M., Nye B.A., Carroll D., Wiffen P.J., Moore R.A. (1996). A systematic review of antidepressants in neuropathic pain. Pain.

[13] Cook R.J., Sackett D.L. (1996). The number needed to treat: A clinically useful measure of treatment effect. BMJ.

[14] Finnerup N.B., Otto M., McQuay H.J., Jensen T.S., Sindrup S.H. (2005). Algorithm for neuropathic pain treatment: An evidence based proposal. Pain.

[15] Pan H.L., Wu Z.Z., Zhou H.Y., Chen S.R., Zhang H.M., Li D.P. (2008). Modulation of pain transmission by, G-protein-coupled receptors. Pharmacol. Ther..

[16] Kimura M., Saito S., Obata H. (2012). Dexmedetomidine decreases hyperalgesia in neuropathic pain by increasing acetylcholine in the spinal cord. Neurosci. Lett..

[17] Paqueron X., Conklin D., Eisenach J.C. (2003). Plasticity in action of intrathecal clonidine to mechanical but not thermal nociception after peripheral nerve injury. Anesthesiology.

[18] Bantel C., Eisenach J.C., Duflo F., Tobin J.R., Childers S.R. (2005). Spinal nerve ligation increases alpha2-adrenergic receptor, G-protein coupling in the spinal cord. Brain Res..

[19] Eisenach J.C., Zhang Y., Duflo F. (2005). Alpha2-adrenoceptors inhibit the intracellular, Ca^2+^ response to electrical stimulation in normal and injured sensory neurons, with increased inhibition of calcitonin gene-related peptide expressing neurons after injury. Neuroscience.

[20] Pan H.L., Chen S.R., Eisenach J.C. (1999). Intrathecal clonidine alleviates allodynia in neuropathic rats: Interaction with spinal muscarinic and nicotinic receptors. Anesthesiology.

[21] Paqueron X., Li X., Bantel C., Tobin J.R., Voytko M.L., Eisenach J.C. (2001). An obligatory role for spinal cholinergic neurons in the antiallodynic effects of clonidine after peripheral nerve injury. Anesthesiology.

[22] Obata H., Li X., Eisenach J.C. (2005). Alpha2-Adrenoceptor activation by clonidine enhances stimulation-evoked acetylcholine release from spinal cord tissue after nerve ligation in rats. Anesthesiology.

[23] Hayashida K., Eisenach J.C. (2010). Spinal alpha2-adrenoceptor-mediated analgesia in neuropathic pain reflects brain-derived nerve growth factor and changes in spinal cholinergic neuronal function. Anesthesiology.

[24] Kim S.H., Chung J.M. (1992). An experimental model for peripheral neuropathy produced by segmental spinal nerve ligation in the rat. Pain.

[25] Baba H., Kohno T., Okamoto M, Goldstein P.A., Shimoji K., Yoshimura M. (1998). Muscarinic facilitation of, GABA release in substantia gelatinosa of the rat spinal dorsal horn. J. Physiol..

[26] Kimura M., Hayashida K., Eisenach J.C., Saito S., Obata H. (2013). Relief of hypersensitivity after nerve injury from systemic donepezil involves spinal cholinergic and γ-aminobutyric acid mechanisms. Anesthesiology.

[27] Ito S., Suto T., Saito S., Obata H. (2017). Repeated administration of duloxetine suppresses neuropathic pain by accumulating effects of noradrenaline in the spinal cord. Anesth. Analg..

[28] Hiroki T., Suto T., Saito S., Obata H. (2017). Repeated administration of amitriptyline in neuropathic pain: Modulation of the noradrenergic descending inhibitory system. Anesth. Analg..

[29] Nakajima K., Obata H., Iriuchijima N., Saito S. (2012). An increase in spinal cord noradrenaline is a major contributor to the antihyperalgesic effect of antidepressants after peripheral nerve injury in the rat. Pain.

[30] Andersen J., Stuhr-Hansen N., Zachariassen L.G., Koldsø H., Schiøtt B., Strømgaard K., Kristensen A.S. (2014). Molecular basis for selective serotonin reuptake inhibition by the antidepressant agent fluoxetine (Prozac). Mol. Pharmacol..

[31] Owens M.J., Knight D.L., Nemeroff C.B. (2000). Paroxetine binding to the rat norepinephrine transporter in vivo. Biol. Psychiatry.

[32] Chen M., Hoshino H., Saito S., Yang Y., Obata H. (2017). Spinal dopaminergic involvement in the antihyperalgesic effect of antidepressants in a rat model of neuropathic pain. Neurosci. Lett..

[33] Baker K.G., Tork I., Hornung J.P., Halasz P. (1989). The human locus coeruleus complex: An immunohistochemical and three dimensional reconstruction study. Exp. Brain Res..

[34] Goldman G., Coleman P.D. (1981). Neuron numbers in locus coeruleus do not change with age in, Fisher 344 rat. Neurobiol. Aging.

[35] Aston-Jones G., Bloom F.E. (1981). Activity of norepinephrine-containing locus coeruleus neurons in behaving rats anticipates fluctuations in the sleep-waking cycle. J. Neurosci..

[36] Berridge C.W., Waterhouse B.D. (2003). The locus coeruleus-noradrenergic system: Modulation of behavioral state and state-dependent cognitive processes. Brain Res. Rev..

[37] Borodovitsyna O., Flamini M., Chandler D. (2017). Noradrenergic modulation of cognition in health and disease. Neural. Plast..

[38] Tsuruoka M., Tamaki J., Maeda M., Hayashi B., Inoue T. (2012). Biological implications of coeruleospinal inhibition of nociceptive processing in the spinal cord. Front. Integr. Neurosci..

[39] Pompeiano O. (1998). Vasopressin in the locus coeruleus and dorsal pontine tegmentum affects posture and vestibulospinal reflexes. Prog. Brain Res..

[40] Szabadi E. (2013). Functional neuroanatomy of the central noradrenergic system. J. Psychopharmacol..

[41] Kimura M., Suto T., Morado-Urbina C.E., Peters C.M., Eisenach J.C., Hayashida K. (2015). Impaired pain-evoked analgesia after nerve injury in rats reflects altered glutamate regulation in the locus coeruleus. Anesthesiology.

[42] Howorth P.W., Teschemacher A.G., Pickering A.E. (2009). Retrograde adenoviral vector targeting of nociresponsive pontospinal noradrenergic neurons in the rat in vivo. J. Comp. Neurol..

[43] Peters C.M., Hayashida K., Suto T., Houle T.T., Aschenbrenner C.A., Martin T.J., Eisenach J.C. (2015). Individual differences in acute pain-induced endogenous analgesia predict time to resolution of postoperative pain in the rat. Anesthesiology.

[44] Matsuoka H., Suto T., Saito S., Obata H. (2016). Amitriptyline, but not pregabalin, reverses the attenuation of noxious stimulus-induced analgesia after nerve injury in rats. Anesth. Analg..

[45] Singewald N., Philippu A. (1998). Release of neurotransmitters in the locus coeruleus. Prog. Neurobiol..

[46] Mateo Y., Fernández-Pastor B., Meana J.J. (2001). Acute and chronic effects of desipramine and clorgyline on alpha(2)-adrenoceptors regulating noradrenergic transmission in the rat brain: A dual-probe microdialysis study. Br. J. Pharmacol..

[47] Grandoso L., Pineda J., Ugedo L. (2004). Comparative study of the effects of desipramine and reboxetine on locus coeruleus neurons in rat brain slices. Neuropharmacology.

[48] Grant M.M., Weiss J.M. (2001). Effects of chronic antidepressant drug administration and electroconvulsive shock on locus coeruleus electrophysiologic activity. Biol. Psychiatry.

[49] Alba-Delgado C., Mico J.A., Sánchez-Blázquez P., Berrocoso E. (2012). Analgesic antidepressants promote the responsiveness of locus coeruleus neurons to noxious stimulation: Implications for neuropathic pain. Pain.

[50] Kajitani N., Hisaoka-Nakashima K., Morioka N., Okada-Tsuchioka M., Kaneko M., Kasai M., Shibasaki C., Nakata Y., Takebayashi M. (2012). Antidepressant acts on astrocytes leading to an increase in the expression of neurotrophic/growth factors: Differential regulation of, FGF-2 by noradrenaline. PLoS ONE.

[51] Reimers J.M., Loweth J.A., Wolf M.E. (2014). BDNF contributes to both rapid and homeostatic alterations in, AMPA receptor surface expression innucleus accumbens medium spiny neurons. Eur. J. Neurosci..

[52] Leventhal L., Smith V., Hornby G., Andree T.H., Brandt M.R., Rogers K.E. (2007). Differential and synergistic effects of selective norepinephrine and serotonin reuptake inhibitors in rodent models of pain. J. Pharmacol. Exp. Ther..

[53] Bardin L. (2011). The complex role of serotonin and 5-HT receptors in chronic pain. Behav. Pharmacol..

[54] Viguier F., Michot B., Hamon M., Bourgoin S. (2013). Multiple roles of serotonin in pain control mechanisms—Implications of 5-HT_7_ and other 5-HT receptor types. Eur. J. Pharmacol..

[55] Yoshimura M., Furue H. (2006). Mechanisms for the anti-nociceptive actions of the descending noradrenergic and serotonergic systems in the spinal cord. J. Pharmacol. Sci..

[56] Millan M.J. (2002). Descending control of pain. Prog. Neurobiol..

[57] Bliss T.V., Collingridge G.L., Kaang B.K., Zhuo M. (2016). Synaptic plasticity in the anterior cingulate cortex in acute and chronic pain. Nat. Rev. Neurosci..

[58] Fields H.L., Heinricher M.M., Mason P. (1991). Neurotransmitters in nociceptive modulatory circuits. Annu. Rev. Neurosci..

[59] Vanegas H., Schaible H.G. (2004). Descending control of persistent pain: Inhibitory or facilitatory?. Brain Res. Brain Res. Rev..

[60] Kuner R. (2010). Central mechanisms of pathological pain. Nat. Med..

[61] Porreca F., Ossipov M.H., Gebhart G.F. (2002). Chronic pain and medullary descending facilitation. Trends Neurosci..

[62] Wei F., Dubner R., Zou S., Ren K., Bai G., Wei D., Guo W. (2010). Molecular depletion of descending serotonin unmasks its novel facilitatory role in the development of persistent pain. J. Neurosci..

[63] Rahman W., Suzuki R., Webber M., Hunt S.P., Dickenson A.H. (2006). Depletion of endogenous spinal 5-HT attenuates the behavioural hypersensitivity to mechanical and cooling stimuli induced by spinal nerve ligation. Pain.

[64] Suzuki R., Rahman W., Hunt S.P., Dickenson A.H. (2004). Descending facilitatory control of mechanically evoked responses is enhanced in deep dorsal horn neurones following peripheral nerve injury. Brain Res..

[65] Bannister K., Patel R., Goncalves L., Townson L., Dickenson A.H. (2015). Diffuse noxious inhibitory controls and nerve injury: Restoring an imbalance between descending monoamine inhibitions and facilitations. Pain.

[66] Avila-Rojas S.H., Velázquez-Lagunas I., Salinas-Abarca A.B., Barragán-Iglesias P., Pineda-Farias J.B., Granados-Soto V. (2015). Role of spinal 5-HT5A, and 5-HT1A/1B/1D, receptors in neuropathic pain induced by spinal nerve ligation in rats. Brain Res..

[67] Okazaki R., Namba H., Yoshida H., Okai H., Miura T., Kawamura M. (2008). The antiallodynic effect of, Neurotropin is mediated via activation of descending pain inhibitory systems in rats with spinal nerve ligation. Anesth. Analg..

[68] Obata H., Saito S., Sasaki M., Ishizaki K., Goto F. (2001). Antiallodynic effect of intrathecally administered 5-HT(2) agonists in rats with nerve ligation. Pain.

[69] Sasaki M., Obata H., Saito S., Goto F. (2003). Antinociception with intrathecal alpha-methyl-5-hydroxytryptamine, a 5-hydroxytryptamine 2A/2C receptor agonist, in two rat models of sustained pain. Anesth. Analg..

[70] Song Z., Meyerson B.A., Linderoth B. (2011). Spinal 5-HT receptors that contribute to the pain-relieving effects of spinal cord stimulation in a rat model of neuropathy. Pain.

[71] Wattiez A.S, Dupuis A., Privat A.M., Chalus M., Chapuy E., Aissouni Y., Eschalier A., Courteix C. (2017). Disruption of 5-HT2A-PDZ protein interaction differently affects the analgesic efficacy of, SSRI, SNRI and, TCA in the treatment of traumatic neuropathic pain in rats. Neuropharmacology.

[72] Sałat K., Kołaczkowski M., Furgała A., Rojek A., Śniecikowska J., Varney M.A., Newman-Tancredi A. (2017). Antinociceptive, antiallodynic and antihyperalgesic effects of the 5-HT1A receptor selective agonist, NLX-112 in mouse models of pain. Neuropharmacology.

[73] Fleetwood-walker S.M., Hope P.J., Mitchell R. (1988). Antinociceptive actions of descending dopaminergic tracts on cat and rat dorsal horn somatosensory neurones. J. Physiol..

[74] Taniguchi W., Nakatsuka T., Miyazaki N., Yamada H., Takeda D., Fujita T., Kumamoto E., Yoshida M. (2011). In vivo patch-clamp analysis of dopaminergic antinociceptive actions on substantia gelatinosa neurons in the spinal cord. Pain.

[75] Hoshino H., Obata H., Nakajima K., Mieda R., Saito S. (2015). The antihyperalgesic effects of intrathecal bupropion, a dopamine and noradrenaline reuptake inhibitor, in a rat model of neuropathic pain. Anesth. Analg..

[76] Morón J.A., Brockington A., Wise R.A., Rocha B.A., Hope B.T. (2002). Dopamine uptake through the norepinephrine transporter in brain regions with low levels of the dopamine transporter: Evidence from knock-out mouse lines. J. Neurosci..

[77] Taylor B.K., Joshi C., Uppal H. (2003). Stimulation of dopamine, D2 receptors in the nucleus accumbens inhibits inflammatory pain. Brain. Res..

[78] Moradi M., Yazdnian M., Haghparast A. (2015). Role of dopamine, D2-like receptors within the ventral tegmental area and nucleus accumbens in antinociception induced by lateral hypothalamus stimulation. Behav. Brain Res..

[79] Wakaizumi K., Kondo T., Hamada Y., Narita M., Kawabe R., Narita H., Watanabe M., Kato S., Senba E., Kobayashi K. (2016). Involvement of mesolimbic dopaminergic network in neuropathic pain relief by treadmill exercise: A study for specific neural control with, Gi-DREADD in mice. Mol. Pain.

[80] Kihara T., Ikeda M. (1995). Effects of duloxetine, a new serotonin and norepinephrine uptake inhibitor, on extracellular monoamine levels in rat frontal cortex. J. Pharmacol. Exp. Ther..

[81] Magnusson J.E., Fisher K. (2000). The involvement of dopamine in nociception: The role of, D(1) and, D(2) receptors in the dorsolateral striatum. Brain Res..

[82] Ansah O.B., Leite-Almeida H., Wei H., Pertovaara A. (2007). Striatal dopamine, D2 receptors attenuate neuropathic hypersensitivity in the rat. Exp. Neurol..

[83] Dick I.E., Brochu R.M., Purohit Y., Kaczorowski G.J., Martin W.J., Priest B.T. (2007). Sodium channel blockade may contribute to the analgesic efficacy of antidepressants. J. Pain.

[84] Sudoh Y., Cahoon E.E., Gerner P., Wang G.K. (2003). Tricyclic antidepressants as long-acting local anesthetics. Pain.

[85] Kalso E. (2005). Sodium channel blockers in neuropathic pain. Curr. Pharm. Des..

[86] Devor M. (2006). Sodium channels and mechanisms of neuropathic pain. J. Pain.

[87] Barygin O.I., Nagaeva E.I., Tikhonov D.B., Belinskaya D.A., Vanchakova N.P., Shestakova N.N. (2017). Inhibition of the, NMDA and, AMPA receptor channels by antidepressants and antipsychotics. Brain Res..

[88] Kohno T., Kimura M., Sasaki M., Obata H., Amaya F., Saito S. (2012). Milnacipran inhibits glutamatergic, *N*-methyl-d-aspartate receptor activity in spinal dorsal horn neurons. Mol. Pain.

[89] Dickenson A.H, Chapman V., Green G.M. (1997). The pharmacology of excitatory and inhibitory amino acid-mediated events in the transmission and modulation of pain in the spinal cord. Gen. Pharmacol..

[90] Herrero J.F., Laird J.M., López-García J.A. (2000). Wind-up of spinal cord neurones and pain sensation: Much ado about something?. Prog. Neurobiol..

[91] Yokogawa F., Kiuchi Y., Ishikawa Y., Otsuka N., Masud Y, Hosoyamada A. (2002). An investigation of monoamine receptors involved in antinociceptive effects of antidepressants. Anesth. Analg..

[92] Antkiewicz-Michaluk L., Romańska I., Michaluk J., Vetulani J. (1991). Role of calcium channels in effects of antidepressant drugs on responsiveness to pain. Psychopharmacology (Berl).

[93] Galeotti N., Ghelardini C., Bartolini A. (2001). Involvement of potassium channels in amitriptyline and clomipramine analgesia. Neuropharmacology.

[94] Phillis J.W., Wu P.H. (1982). The effect of various centrally active drugs on adenosine uptake by the central nervous system. Comp. Biochem. Physiol..

[95] McCarson K.E., Duric V., Reisman S.A., Winter M., Enna S.J. (2006). GABA(B) receptor function and subunit expression in the rat spinal cord as indicators of stress and the antinociceptive response to antidepressants. Brain Res..

[96] Isenberg K.E., Cicero T.J. (1984). Possible involvement of opiate receptors in the pharmacological profiles of antidepressant compounds. Eur. J. Pharmacol..

[97] Yaron I., Shirazi I., Judovich R., Levartovsky D., Caspi D., Yaron M. (1999). Fluoxetine and amitriptyline inhibit nitric oxide, prostaglandin, E2, and hyaluronic acid production in human synovial cells and synovial tissue cultures. Arthritis Rheumatol..

